# Relayed hyperpolarization from *para*-hydrogen improves the NMR detectability of alcohols†
†Electronic supplementary information (ESI) available: Experimental methods, NMR spectra. See DOI: 10.15124/db5b8475-8c71-4714-95f7-eb5e2a49632c


**DOI:** 10.1039/c9sc02765c

**Published:** 2019-07-01

**Authors:** Peter J. Rayner, Ben. J. Tickner, Wissam Iali, Marianna Fekete, Alastair D. Robinson, Simon B. Duckett

**Affiliations:** a Centre for Hyperpolarisation in Magnetic Resonance , Department of Chemistry , University of York , Heslington , YO10 5DD , UK . Email: simon.duckett@york.ac.uk

## Abstract

The detection of alcohols by magnetic resonance techniques is important for their characterization and the monitoring of chemical change. Hyperpolarization processes can make previously inpractical measurements, such as the determination of low concentration intermediates, possible. Here, we investigate the SABRE-Relay method in order to define its key characteristics and improve the resulting ^1^H NMR signal gains which subsequently approach 10^3^ per proton. We identify optimal amine proton transfer agents for SABRE-Relay and show how catalyst structure influences the outcome. The breadth of the method is revealed by expansion to more complex alcohols and the polarization of heteronuclei.

## Abstract 2

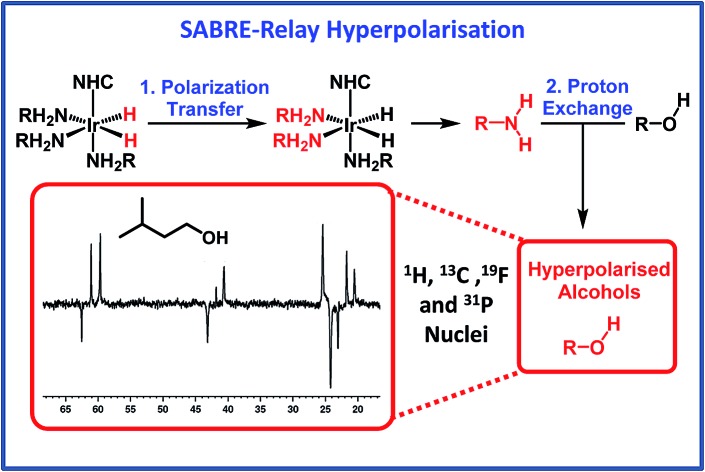
NMR signal strength improvements delivered through hyperpolarisation *via para*-hydrogen enable the facile detection of alcohols.

## 

Molecules that contain an alcohol functional group are present widely in materials used in industrial, biochemical and medical applications. Their complexity can vary from simple structures such as ethanol to polyfunctional macromolecules such as DNA and carbohydrates. The solution state characterization and quantification of these materials commonly involves Nuclear Magnetic Resonance (NMR) spectroscopy and, to a lesser extent, Magnetic Resonance Imaging (MRI). MR techniques have the benefit of being non-invasive and do not require ionizing radiation thereby securing their clinical importance. However, both NMR and MRI suffer from low sensitivity as their underlying signal strength is derived from the population difference that exists between two distinct nuclear spin orientations that are close in energy.

The perturbation of the spin state populations of these energy levels away from the Boltzmann distribution can be achieved using methods that are collectively known as hyperpolarization. Dynamic Nuclear Polarization (DNP) is one of the most well-developed of these techniques and has been applied to the study of disease through the *in vivo* monitoring of biomolecule metabolism.^1^–^7^ Additionally, Spin Exchange Optical Pumping of noble gas nuclei has proven successful in producing diagnostic high resolution images of lung pathologies.^8^,^9^

A related technique, termed *Para*-Hydrogen Induced Polarization (PHIP), which has provoked a significant amount of attention, derives its non-Boltzmann spin distribution from *para*-hydrogen (*p*-H_2_) gas. Its two nuclear spins are aligned anti-parallel in a singlet state and were predicted to deliver strong ^1^H NMR signals when added to an unsaturated material.^10^,^11^ Experimentally, this hypothesis has since been validated many times and the resulting enhanced signals have been used for the examination of numerous reaction mechanisms^12^–^18^ and for the determination of low concentration intermediates.^19^–^21^ A major barrier to the widespread application of this approach is the requirement that an unsaturated *p*-H_2_ acceptor is needed to create a magnetic environment where the singlet symmetry of *p*-H_2_ is broken and hence allows the products of chemical change to be detected.^22^–^25^ One route to overcome this challenge has involved the application of cleavable unsaturated molecular tags which has ultimately allowed the hyperpolarization of pyruvate, acetate and lactate and the subsequent monitoring of metabolism.^26^–^28^

Alternatively, it is possible to hyperpolarize a range of molecules using *p*-H_2_ without chemical modification through the Signal Amplification By Reversible Exchange process (SABRE).^29^,^30^ This requires the simultaneous and reversible binding of the substrate and *p*-H_2_ derived hydrogen atoms to a suitable reaction center. The spin–spin couplings^31^ that result between their NMR active nuclei allows spontaneous hyperpolarization transfer to occur at low-field.^32^–^35^ Upon substrate dissociation, the NMR resonances of these chemically unchanged materials become strongly enhanced. A growing range of substrates have proven to be amenable to the SABRE polarization method that typically contain a nitrogen heterocycle,^29^ nitrile,^36^ diazirine^37^,^38^ or amine^39^ functionality with their ^1^H and X-nuclei being sensitized.^30^,^40^

More recently, the SABRE substrate scope has been extended to include poorly ligating molecules that contain a labile proton.^41^ In this development, termed SABRE-Relay (Scheme 1), the initial SABRE polarization of an amine enables a hyperpolarized proton to be transferred into the target analyte *via* proton exchange. Subsequently, the resulting enhanced nuclear spin population difference can be seen in the analyte's NMR active nuclei. When this process is conducted in the presence of just 1 μL of an alcohol, ^1^H and ^13^C NMR signal gains of up to 650-fold and 600-fold respectively have been reported in the single scan NMR spectra of the straight chain alcohols methanol through octanol at 9.4 T.^41^ Hence, the analytical potential of this method for the detection of alcohols at low concentrations is clear.

**Scheme 1:**
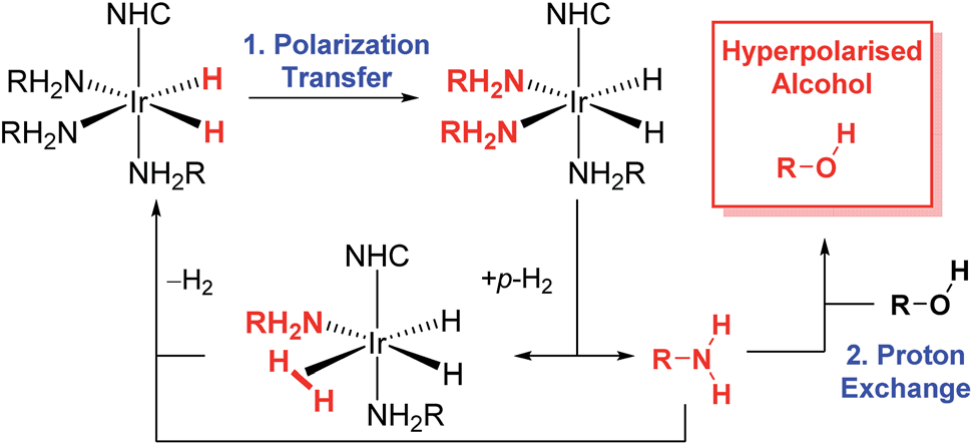
The SABRE-Relay catalytic cycle for hyperpolarization for alcohols. Polarization is initially transferred from the *p*-H_2_ derived hydride ligands to a carrier amine (RNH_2_) which subsequently transfer polarization to the alcohol *via* proton exchange. NHC is N-heterocyclic carbene.

We seek here to probe the underlying processes that govern SABRE-Relay and control them for the optimal ^1^H and heteronuclear NMR detection of alcohols. We begin by using 1-propanol as a test analyte prior to increasing substrate complexity by introducing secondary and tertiary alcohols and other functional groups. A number of kinetic factors are observed to be central to the multi-step SABRE-Relay technique. First, it is well established that the lifetime of active catalyst is instrumental in controlling SABRE polarization transfer efficiency.^35^ There are a number of methods for controlling this parameter including ligand design^42^,^43^ and temperature variation. The optimal catalyst lifetime is related to the size of its hydride–hydride and hydride–substrate scalar coupling constants.^32^,^35^,^44^ The second consideration for SABRE-Relay is the rate of proton transfer between the SABRE hyperpolarized amine and the target alcohol. It might be expected that their relative p*K*_a_ values should be important and thus the identity of the amine could be critical for achieving large NMR signal gains. It has been previously shown that primary amines themselves achieve good levels of SABRE polarization, with up to 1000-fold enhancement in the NH proton responses of *d*_7_-BnNH_2_ being quantified at 9.4 T.^39^ Additionally, aromatic amines, such as imidazole, also undergo efficient SABRE transfer and could therefore be potential hyperpolarization carriers.^45^ We now set out to examine these effects.

## Role of carrier amine

**Fig. 1:**
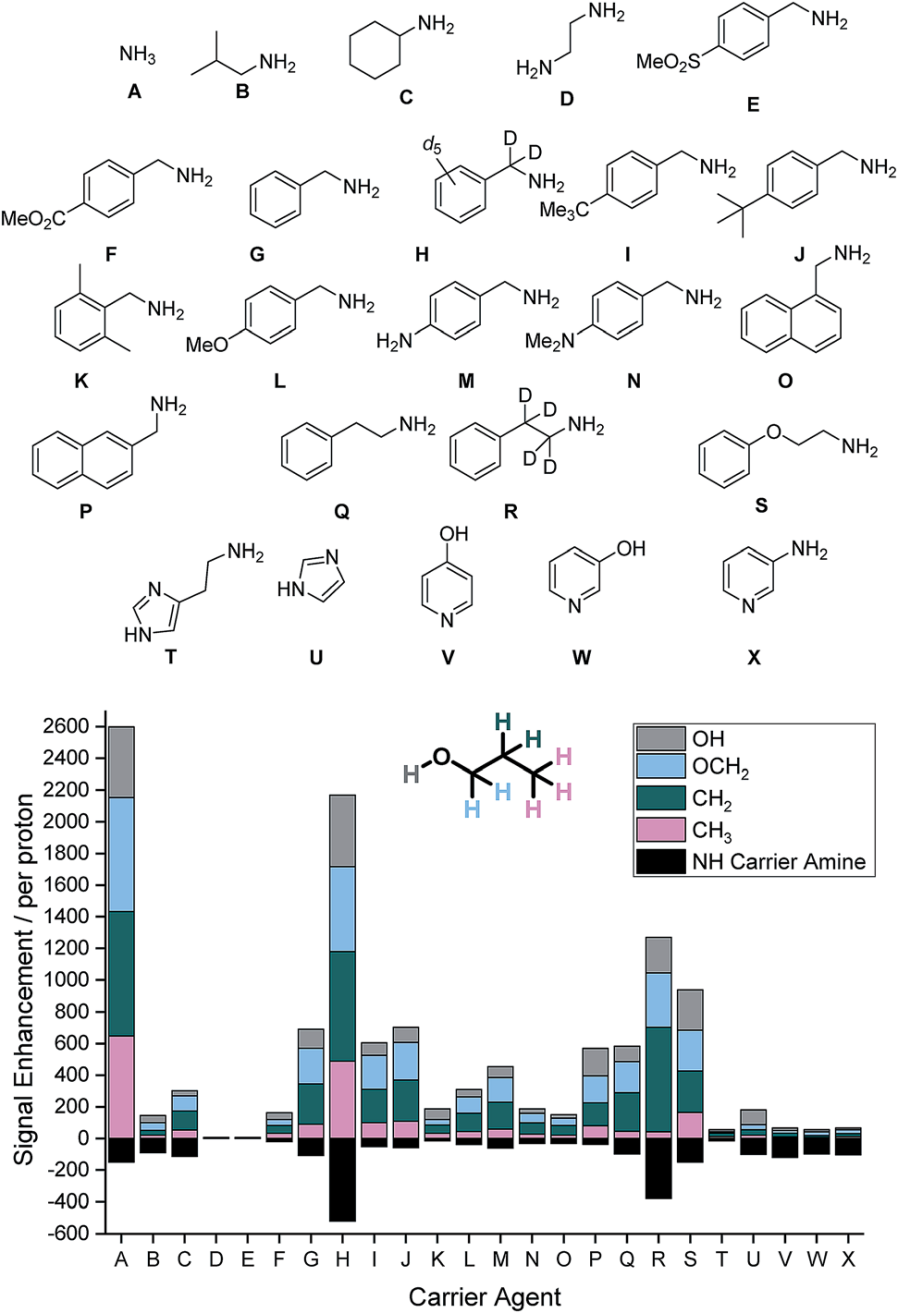
(Top) Structures of carrier agents **A–X** screened for SABRE-Relay polarization of 1-propanol and the resulting ^1^H NMR signal enhancements within the spin system per proton at 9.4 T they achieve (Bottom). Enhancements for each proton environment are characterized by the height of each individual colour bar.

Our study begins with an examination of the 24 hyperpolarization carriers shown in Fig. 1. Multiple samples containing [IrCl(COD)(IMes)] (5 mM), the amine (**A–X**, 10 eq.), 1-propanol (1 μL) and dichloromethane-*d*_2_ (0.6 mL) were prepared and then exposed to 3 bar H_2_ for 16 h at room temperature to form the active SABRE catalyst [Ir(H)_2_(IMes)(**A–X**)_3_]Cl. These samples were then shaken with 3 bar *p*-H_2_ for 10 seconds at 70 G prior to rapid transfer into a 9.4 T field for interrogation by NMR spectroscopy. The resulting ^1^H NMR signal enhancements for each proton environment in 1-propanol were then calculated. These values are presented per proton graphically in Fig. 1 alongside the NH enhancement levels for the carrier amines in the absence of 1-propanol.

SABRE-Relay conditions that use ammonia (**A**) as the carrier gave the largest signal enhancements for 1-propanol. We quantified enhancements of 441, 723, 783 and 648-fold per proton in the O*H*, OC*H*_2_, C*H*_2_ and C*H*_3_ positions respectively. In contrast, isobutylamine (**B**) gave comparably poor signal enhancements of less than 50-fold per proton environment. When cyclohexylamine (**C**) was employed the signal gains were slightly improved relative to **B** (*ca.* 50–120-fold), however, ethylenediamine (**D**) resulted in no polarization transfer to 1-propanol. This is due to the formation of a stable complex as the result of bidentate binding of the diamine which prevents ligand exchange. Such methods have previously been used to deactivate the SABRE catalyst and return substrate *T*_1_ values to their usual value in the absence of the SABRE catalyst.^46^

The carrier amines **E–P** all contain the benzylamine motif, however the substituents on the aromatic ring differ. Firstly, 4-methylsulfonylbenzyl amine (**E**) does not exhibit polarization transfer to 1-propanol due an active SABRE catalyst not being formed. The methyl ester derivative (**F**) does facilitate SABRE-Relay polarization transfer, however the signal gains now reach a maximum of 48-fold for the OC*H*_2_ resonance. Benzylamine (**G**) performs better with signal enhancements of 225-fold for the OC*H*_2_. This result can be significantly improved upon through the use of the deuterated isotopologue, *d*_7_-benzylamine (**H**) which now gives enhancements of 452, 537, 690 and 489-fold for the O*H*, OC*H*_2_, C*H*_2_ and C*H*_3_ positions respectively. Introduction of either a methyl (**I**) or *tert*-butyl (**J**) group on the aromatic ring gave comparable results to that of benzylamine and we would therefore expect a similar increase in SABRE-Relay performance on deuteration. The introduction of the electron donating groups methoxy (**L**), amino (**M**) and dimethylamino (**N**) all reduced the efficacy of polarization transfer to 1-propanol despite exhibiting consistent NH signal gains.

Regioisomeric naphthyl derivatives, 1-napthylmethyl amine (**O**) and 2-napthylmethyl amine (**P**), also facilitate SABRE-Relay polarization of 1-propanol. **P** though gives significantly improved performance when compared to **O**. We attribute this to the improved polarization of the NH proton in the carrier amine whose enhancements are now *ca.* 3-fold greater for **P**, an effect which is likely to be due to steric differences in the regioisomers. For the case of phenylethylamine (**Q**), where a C*H*_2_ spacer is added into the aliphatic chain, the signal gains of 1-propanol are slightly below those of benzylamine. Deuteration of the aliphatic portion of this carrier amine (**R**) again improves the polarization levels in the alcohol and highlights the importance that isotopic labelling has on the SABRE-Relay outcome. This increase is attributed to a corresponding increase in the NH signal gain of the free amine (108 compared to 391-fold per proton for **Q** and **R** respectively). Introducing an ether linkage to give phenoxyethylamine (**S**) also improves the signal gains seen in 1-propanol above those with benzylamine. Now the signal enhancements were quantified to be 255, 258, 261 and 165-fold for the O*H*, OC*H*_2_, C*H*_2_ and C*H*_3_ resonances respectively. This is the best performing of the organic amines tested that does not contain deuterium; we would expect its SABRE-Relay enhancement to further improve if it were ^2^H-labelled. However, despite a number of synthetic methods being employed to yield the deuterated variant, we were unable to isolate the desired product with high isotopic enrichment.

Finally, a number of aromatic amines were screened for SABRE-Relay transfer, however, they all showed disappointing signal gains for 1-propanol. Interestingly, all of these carrier amines showed good signal gains for their NH resonances. Consequently, we propose that the necessary NH ↔ OH proton transfer step no longer occurs on an appropriate timescale to efficiently mediate the transfer of polarization into 1-propanol.

The optimum amine SABRE-Relay transfer agents for the polarization of propanol in this series were therefore NH_3_ (**A**) and *d*_7_-BnNH_2_ (**F**). This is despite the fact that the raw SABRE signal enhancement seen per NH proton in NH_3_ is worse (*ca.* 150-fold) than that of *d*_7_-BnNH_2_ (*ca.* 570-fold). Furthermore, the NH protons of NH_3_ exhibit a *T*_1_ relaxation time of just 5.5 s in dichloromethane-*d*_2_ at 9.4 T while those of *d*_7_-BnNH_2_ are 10.1 s.^39^ Hence, it is postulated that NH ↔ OH proton exchange between NH_3_ and 1-propanol must proceed on a more favorable timescale for polarization transfer than the analogous process between *d*_7_-BnNH_2_ and 1-propanol. We note that for both carrier agents, this exchange rate is too rapid for measurement by EXSY, even at reduced temperatures, and that the conjugate acids of NH_3_ and BnNH_2_ have very close p*K*_a_ values of 9.21 and 9.34 respectively in H_2_O.^47^ However, *d*_7_-BnNH_2_ does have one significant advantage over the use of NH_3_ as the SABRE-Relay carrier amine because it is a liquid at room temperature. Therefore, it can be accurately measured into these samples whereas the handling of gaseous NH_3_ is more challenging.

## Effect of contaminant H_2_O

During the course of these hyperpolarization measurements, it was noted that the presence of residual H_2_O in the sample, originating from the solvent, alcohol or amine, dramatically affects the resulting signal gains. To quantify this effect, a sample containing [IrCl(COD)(IMes)], *d*_7_-BnNH_2_ (10 eq.) and 1-propanol (1 μL) in dichloromethane-*d*_2_ was doped with 1 and 5 μL of H_2_O. This resulted in the observed NMR signal enhancements falling dramatically from 537-fold for the OC*H*_2_ resonance to 48 and 11-fold respectively. In addition, an NMR signal of growing strength is seen for hyperpolarized H_2_O in these samples. This change is due to a combination of increased spin-dilution, as the finite *p*-H_2_ polarization reservoir is shared with an increased number of spins and a reduced efficacy in polarization transfer between the amine and the alcohol. For this reason, we conclude that carrying out SABRE-Relay under anhydrous conditions, a process that can be readily achieved by distillation of the solvent from CaH_2_, is beneficial.^48^

## Effect of amine and alcohol concentration

SABRE derived signal enhancements are known to be highly dependent on the ratio of catalyst to substrate with lower concentrations typically yielding higher signal gains.^49^,^50^ It is accepted that this is a consequence of the available *p*-H_2_ derived polarization pool being shared across a finite number of spins. Co-ligands have therefore been employed to reduce spin-dilution and often provide improved signal gains.^43^,^51^ For SABRE-Relay, we propose that it is not only the total signal enhancement of the carrier amine but the efficiency of constructive proton exchange between the amine and the alcohol that is important. To probe these two effects a number of dry samples were prepared that contained increasing concentrations of *d*_7_-BnNH_2_ and alcohol relative to the [IrCl(COD)(IMes)] pre-catalyst. We chose to focus our attention on the amine *d*_7_-BnNH_2_ due to the ability to accurately vary the amount of amine added.

First, the effect of the amine concentration was determined by increasing the number of *d*_7_-BnNH_2_ equivalents relative to iridium from 5 to 25 in the presence of 1 μL of 1-propanol. This study showed that the highest signal gains seen for the OC*H*_2_ resonance of 1-propanol were observed when between 5–8 equivalents *d*_7_-BnNH_2_ was employed and this corresponds to an amine concentration of 25–40 mM (see ESI†). For example, at 5 equivalents of *d*_7_-BnNH_2_ a 718-fold signal gain for the OC*H*_2_ was recorded whereas at 8 equivalents a comparable signal gain of 695-fold was quantified. The OC*H*_2_ signal gain decreases to *ca.* 58-fold with the highest amine concentration of 125 mM.

Second, the effect of alcohol concentration was determined in a similar fashion by varying the volume of 1-propanol between 0.1–7.0 μL (0.4–30 eq. based on iridium) whilst maintaining a fixed 5 eq. of *d*_7_-BnNH_2_ and a 5 mM concentration of [IrCl(COD)(IMes)]. At low relative concentrations of alcohol, the OC*H*_2_ signal gain was reduced. For example, when just 0.4 eq. (2 mM) of 1-propanol was present in the SABRE-Relay catalysis, a signal gain of 128-fold was quantified. As the relative amount of alcohol is increased to 5 equivalents, the OC*H*_2_ signal gain increases to reach a maximum of 723-fold per proton. Increasing the alcohol concentration further then leads to a decrease in signal gain.

These observations confirm that the relative rate of NH ↔ OH exchange is important. Based on the literature, it is likely to be bimolecular in nature, proceeding through an [R-NH_3_^+^][^–^O-R′] type intermediate.^52^,^53^ When the alcohol is present at low concentration, NH ↔ NH exchange between the carrier amine dominates. This reduces the proliferation of polarization to 1-propanol and lower signal gains result. At the higher loadings of 1-propanol, either NH ↔ OH and OH ↔ OH exchange is too rapid for efficient low field polarization or there is a reduction based on the increasing number of protons relative to those in *p*-H_2_ which are limited in accordance with the volume of gas in the NMR tube. Therefore, working with higher pressures of *p*-H_2_ could be expected to further increase the size of the hyperpolarized signals. It is possible to conclude here that for 1-propanol, the largest relayed NMR signal enhancements are achieved when using equimolar amounts of alcohol and carrier amine.

## Influence of polarization transfer field

**Fig. 2:**
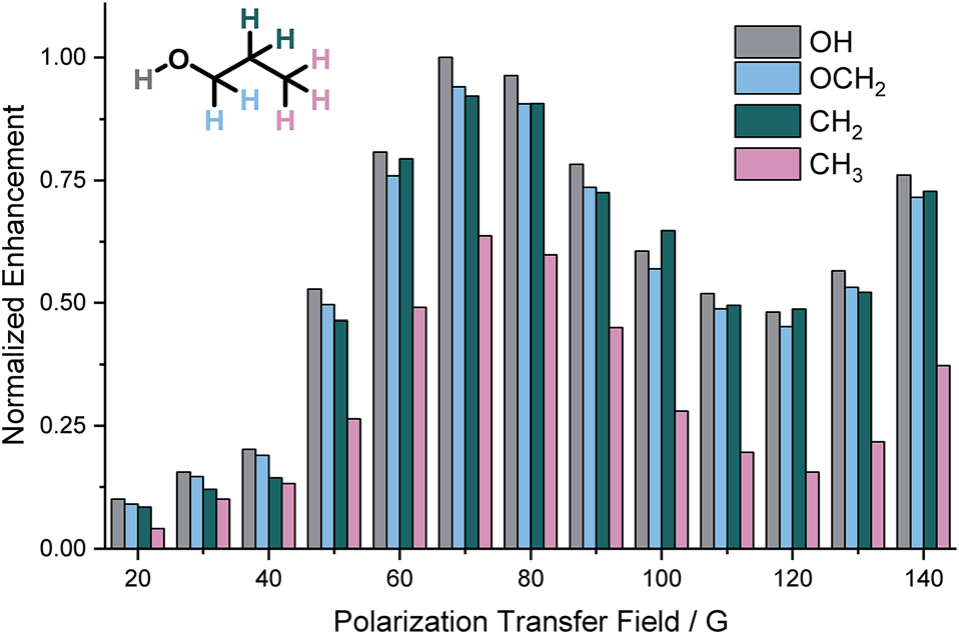
Normalized ^1^H NMR signal enhancements in propanol achieved using BnNH_2_ as the SABRE-Relay agent as a function of the size of the polarization transfer field.

For SABRE, the most efficient polarization transfer is observed at the magnetic level anti-crossing point^54^ where the size of the hydride–hydride coupling matches optimally with the difference between precession frequencies of hydride and substrate nuclei.^32^,^44^ However, for SABRE-Relay, whilst the hydride–hydride coupling in the *tris*-amine complex of the type [Ir(H)_2_(IMes)(amine)_3_]Cl will determine the optimal polarization field for the carrier amine polarization while transfer within the alcohol will be governed by spin–spin couplings between the OH and the aliphatic chain. Therefore, the field dependence on the observed polarization levels was probed over the range 20 to 140 G using an automated NMR flow system.^55^ Due to the volatility of NH_3_ and dichloromethane-*d*_2_, we carried out these experiments using BnNH_2_ with CDCl_3_ as solvent. The relative signal enhancements from these measurements are shown in Fig. 2. The most efficient polarization transfer was observed at 70 G which is optimal for polarization transfer utilizing the hydride–NH ^3^*J* coupling within the active SABRE catalyst. Hence, it would seem that the initial SABRE polarization transfer step is critical to this process. Interestingly, as the transfer field increases above 130 G a growth in NMR signal enhancement is observed which reflects the limit of our equipment.

## Catalyst identity

**Fig. 3:**
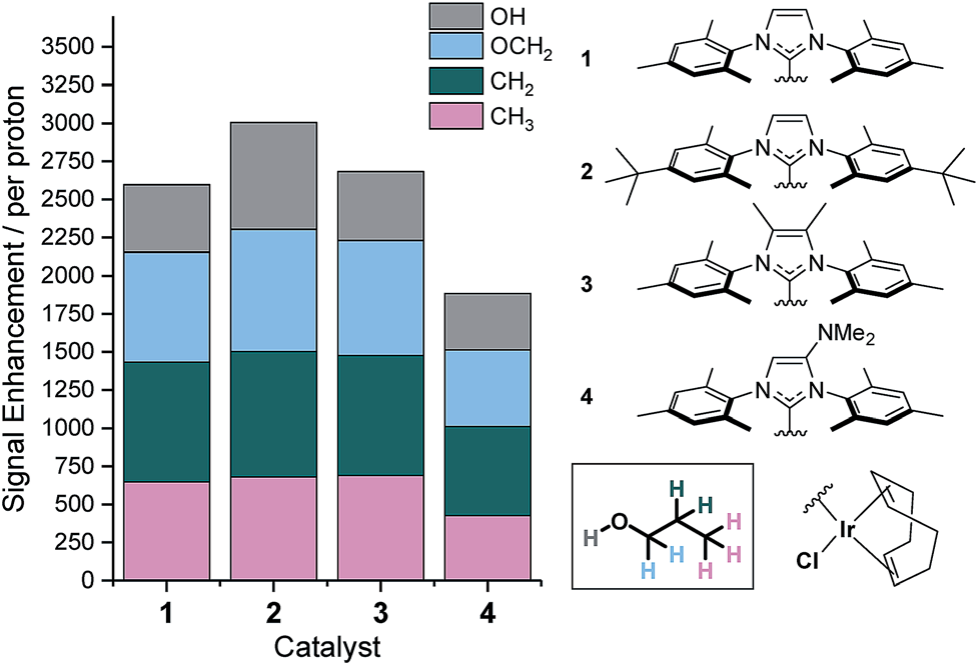
Structures of catalysts **1–4** and the resulting ^1^H NMR signal enhancements per proton they deliver to 1-propanol at 9.4 T after SABRE-Relay transfer from NH_3_. Enhancements for each proton environment are characterized by the height of each individual colour bar.

We have previously shown that the rate of NH_3_ ligand loss from [Ir(H)_2_(IMes)(NH_3_)_3_]Cl is just 1.64 s^–1^ at 298 K (ref. 39) and therefore lower than that predicted^34^ to be optimal. One option to increase this dissociation rate is to warm the samples and thus, at 308 K the resulting SABRE induced signal gain of NH_3_ increases from 154-fold to 251-fold. However, an alternative method to modulate the rate of substrate dissociation is *via* changes to the catalysts' N-heterocyclic carbene ligand.^43^ As such we chose to compare the IMes derived catalyst to one with *tert*-butyl substituents on the aryl arms of the NHC and one with methyl groups on the imidazole ring (catalysts **2** and **3** of Fig. 3). Additionally, we prepared a further electron rich NHC that bears an NMe_2_ group on the imidazole ring (catalyst **4**), which has previously been shown to increase the efficacy of palladium catalyzed Buchwald–Hartwig aminations.^56^ To the best of our knowledge this highly electron rich catalyst has not been used for SABRE polarization transfer before. Samples containing [IrCl(COD)(NHC)] (**1–4**, 5 mM), NH_3_ (6–8 eq.) and propanol (1 μL) in anhydrous dichloromethane-*d*_2_ (0.6 mL) were exposed to 3 bar *p*-H_2_ and shaken in a 70 G field. Signal enhancements per proton were then quantified for each catalyst system as detailed in Fig. 3.

It is apparent that catalyst **2** improves the observed NMR signal enhancements for 1-propanol when compared to those achieved with the IMes derived catalyst, **1**. Consequently, an increase in NMR signal gain for each aliphatic site is observed; for example the OC*H*_2_ signal gain improves from 783-fold with **1** to 821-fold with **2** at 9.4 T. However, the O*H* polarization level actually increases far more dramatically from 442 to 701-fold which reflects a *ca.* 70% increase. These signal gain increases are attributed to the resulting increase in rate of NH_3_ dissociation from the active catalyst which is now 3.20 s^–1^ at 298 K and approximately double that seen for **1**. Further, small improvements in these signal enhancement levels were observed when using the deuterated isotopologue of **2**, ***d*_34_-2**.^43^ In this case, the signal gains per proton are now 721, 843, 820, 691-fold for the O*H*, OC*H*_2_, C*H*_2_ and C*H*_3_ positions respectively. Intriguingly, these relayed changes are less significant than for substrates that undergo direct SABRE polarization, where increases of up to 150% have been seen when using a deuterated NHC instead of its protio counterpart.^51^ To investigate this behavior, we measured the *T*_1_ relaxation times of the NH resonance in NH_3_ whilst in the presence of the active catalysts formed from **2** and ***d*_34_-2** under 3 bar H_2_. The *T*_1_ relaxation time is slightly extended from 5.31 to 5.64 s when the deuterated isotopologue is used. This extension is just 6% and significantly smaller than that typically observed for substrates which participate in direct SABRE transfer and may account for the corresponding reduced polarization increases seen during SABRE-Relay.^43^,^51^

**Fig. 4:**
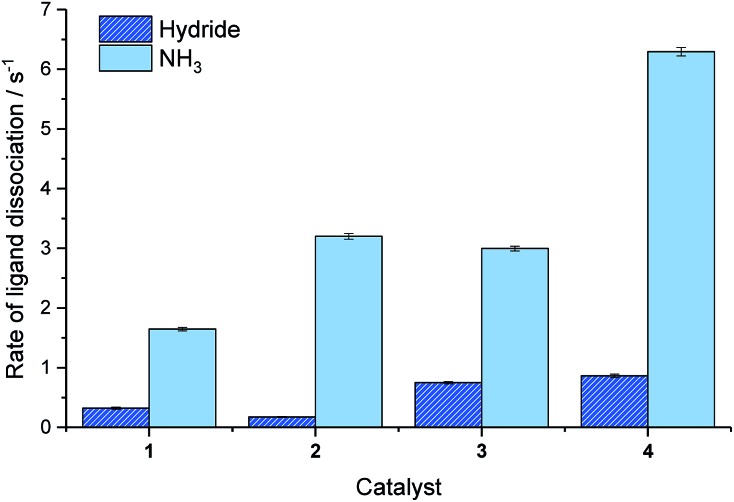
Effective rates of ligand dissociation (s^–1^) from the corresponding SABRE catalyst at 298 K as measured by EXSY spectroscopy.

Catalyst **3** also gave modest improvements in NMR signal gain when compared to **1** but less than those of **2**. For this ligand scaffold, the rate of NH_3_ dissociation from the active catalyst is 2.99 s^–1^ at 298 K and therefore comparable to **2** (Fig. 4). However, now the rate of hydride ligand loss to form H_2_ is more than doubled from 0.32 s^–1^ for **1** to 0.75 s^–1^ for **3**. This increase will cause more rapid *p*-H_2_ consumption and could result in less efficient SABRE as the amount of *p*-H_2_ present within the experiment is finite. In contrast, the rate of hydride loss from the catalyst derived from **2** is lower at 0.17 s^–1^. Lower signal enhancements are observed with **4** when compared to **1–3**. For this system, the rate of dissociation of NH_3_ and the hydride ligands from the active catalyst is significantly higher with values of 6.29 s^–1^ and 0.86 s^–1^ recorded respectively at 298 K. This suggests that the new catalyst lifetime is less than optimal for efficient SABRE transfer.^34^,^43^

## Introducing structural complexity

### ^1^H-signal gains under SABRE-Relay

**Fig. 5:**
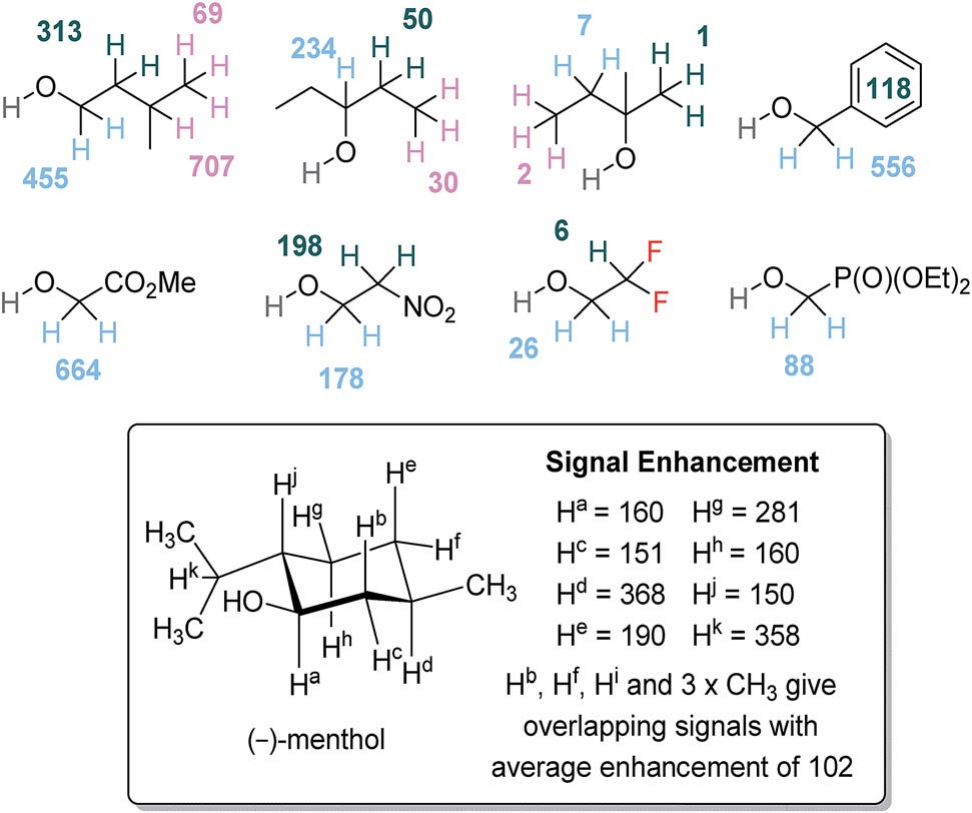
^1^H NMR per proton signal gains for structurally diverse alcohols at 9.4 T. NMR assignments of (–)-menthol were made by comparison to literature data.^57^

To further understand the SABRE-Relay method, structurally complex alcohols were examined, including secondary and tertiary alcohols alongside other OH containing materials, as shown in Fig. 5. First, the regioisomeric alcohols 3-methyl-1-butanol, 3-pentanol and 2-methyl-2-butanol were polarized using the optimized SABRE-Relay conditions for propanol (5 mM of **1**, 7 equivalents of ammonia and 5 equivalents of alcohol in dry dichloromethane-*d*_2_). 3-Methyl-1-butanol gave good ^1^H signal gains with the OC*H*_2_ signal now being 455-fold larger than the signal produced under Boltzmann conditions which is comparable to that of 1-propanol. Polarization is also proliferated throughout the aliphatic chain, with signal gains of 313, 707 and 69-fold being quantified for the C*H*_2_, C*H* and C*H*_3_ groups respectively.

When the secondary alcohol 3-pentanol was investigated under the same conditions, the ^1^H NMR resonance for OC*H* showed a 234-fold signal gain. However, SABRE-Relay enhancements of 50 and 30-fold were seen for the *β* and *γ*-positions. Poor SABRE-Relay performance is observed for the tertiary alcohol 2-methyl-2-butanol, for which ^1^H signal gains of just 1–7-fold could be quantified. Interestingly, the O*H* NMR signal enhancements were comparable for both of these materials (between 200 and 300-fold). Therefore, we conclude that a limiting factor in SABRE-Relay polarization transfer is propagation through the alcohol's scalar coupling network from the OH group. The tension between optimal SABRE polarization of the carrier amine and subsequent proliferation of polarization after proton exchange is thus highlighted.

Benzyl alcohol gave good ^1^H NMR signal enhancements for the OC*H*_2_ site, giving a 556-fold per proton gain while those protons of the phenyl ring achieved an average signal gain of 118-fold per proton. Other structurally diverse alcohols such as methyl glycolate and 2-nitroethanol also give good ^1^H signal enhancement for their aliphatic OC*H*_2_ resonance of 664-fold and 178-fold respectively. Finally, we investigated the natural product (–)-menthol which contains three stereogenic centers and fourteen distinct proton environments. After SABRE-Relay polarization transfer using NH_3_ as the carrier amine we were able to detect ^1^H signal enhancements in each of the protons with up to 368-fold signal gain being quantified.

### ^13^C-signal gains under SABRE-Relay

SABRE-Relay derived polarization can also be transferred to the ^13^C nuclei within the target molecule.^41^ For our test substrate, 1-propanol, we are able to achieve signal gains of 281, 342 and 128-fold for the O*C*H_2_, *C*H_2_ and *C*H_3_ positions respectively when using the previously optimised conditions for ^1^H polarisation (5 mM of ***d*_34_-2**, 5 equivalents of NH_3_ and 5 equivalents of 1-propanol). These signal gains are sufficient to obtain a fully diagnostic ^13^C NMR spectrum in a single scan after spontaneous polarisation transfer at 70 G to the carbon nuclei that are present at their natural isotopic abundance for a sample containing 25 mM of 1-propanol.

**Fig. 6:**
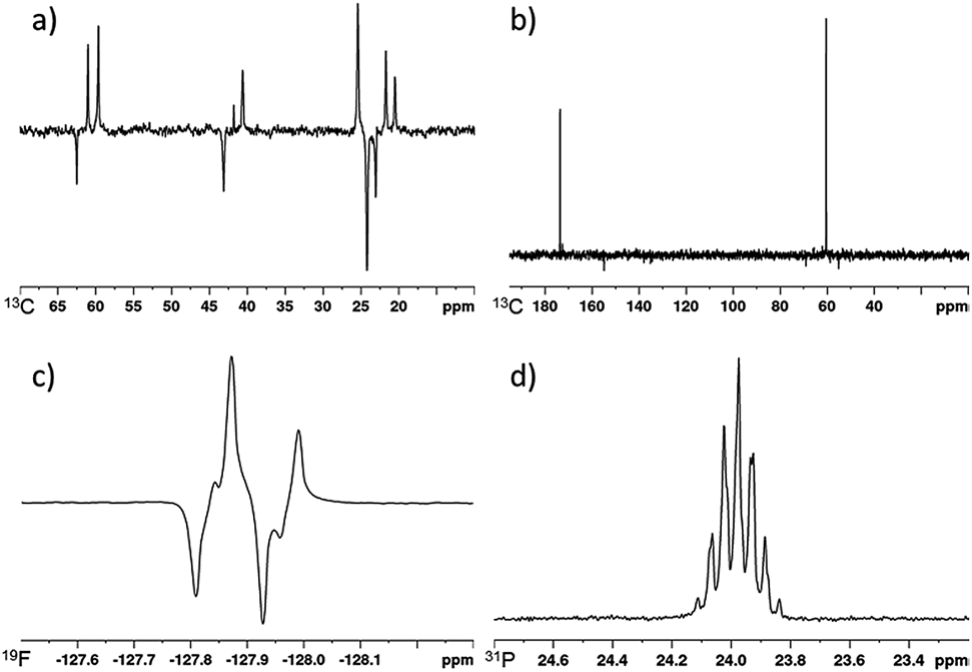
(a) SABRE-Relay hyperpolarized ^13^C spectrum of 3-methyl-1-butanol, (b) SABRE-Relay hyperpolarized ^1^H–^13^C spectrum of methyl glycolate, (c) SABRE-Relay hyperpolarized ^1^H–^19^F INEPT spectrum of 2,2-difluoroethanol, (d) SABRE-Relay hyperpolarized ^31^P spectrum of diethyl (hydroxymethyl) phosphonate. Corresponding thermally polarised spectra are available in the ESI.†

The effect of SABRE-Relay polarization into the ^13^C nuclei in more structurally complex alcohols was also investigated. For 3-methyl-1-butanol, the signals for all its ^13^C sites are also readily observed in the corresponding NMR spectrum after spontaneous polarization transfer at 70 G (Fig. 6a). Now the signal gains were quantified to be 1404 and 1090-fold for the O*C*H_2_ and *C*H_2_ resonances respectively and an average of 403-fold being seen across the overlapping *C*H and *C*H_3_ signals. The signal to noise ratio in this NMR spectrum that was collected at 9.4 T on a 5 mm inverse probe was 18 for the O*C*H_2_ resonance. We note that the conditions required to achieve the highest ^1^H signal gains for 3-methyl-1-butanol discussed earlier also result in the highest ^13^C signal gains. When the concentration of alcohol or carrier is varied from these optimal conditions, the corresponding ^13^C signal gains decrease in the same fashion as the ^1^H signal enhancements (see ESI†). We also note that for 3-methyl-1-butanol, the use of NH_3_ (**A**) as a carrier is necessary to observe SABRE-Relay hyperpolarization in the ^13^C responses in a single scan. When *d*_7_-BnNH_2_ (**H**) is utilized for this alcohol, no hyperpolarised ^13^C resonances are detectable in a single scan. We attribute this to significantly reduced polarization transfer to 3-methyl-1-butanol which is also reflected in weak ^1^H polarisation levels observed under the same conditions (96, 77, 187 and 56-fold for the OC*H*_2_, C*H*_2_, C*H* and C*H*_3_ respectively). These values are significantly lower than those achieved using **A**.

When the ^13^C SABRE-Relay polarization of 3-pentanol was investigated using **A** as the carrier, significantly lower signal gains were recorded when compared to 3-methyl-1-butanol (67, 0 and 38-fold for *C*H, *C*H_2_ and *C*H_3_ positions respectively). We hypothesize that this is likely to be due to inefficient proliferation of the polarization through the alcohol's scalar coupling network at the polarization transfer field. However, by using a ^1^H–^13^C INEPT sequence at high field the signal to noise ratio of the alcohol's ^13^C response can be improved from 3 to 8. Contrastingly, no ^13^C signals are visible for the tertiary alcohol 2-methyl-2-butanol after either spontaneous polarization transfer or using a ^1^H–^13^C INEPT sequence which is consistent with its limited ^1^H performance.

### ^19^F and ^31^P signal gains under SABRE-Relay

The SABRE-Relay polarization of other heteronuclei in molecules that contain an alcohol functional group was also explored. When 2,2-difluoroethanol was used as the target alcohol, SABRE-Relay transfer could be observed into both the ^1^H and ^19^F spins. The OC*H*_2_^1^H-signal gains were just 26-fold and an 11-fold signal gain in the ^19^F spectrum was observed after spontaneous transfer at 70 G during the SABRE-Relay process. The observed ^19^F signal could be improved by using a ^1^H–^19^F INEPT sequence and now a 63-fold enhancement was quantified (Fig. 6c). This improvement is likely to be a combination of inefficient transfer into the ^19^F nuclei at the polarization transfer field and a reflection of the longer ^1^H *T*_1_ values (18.9 and 24.6 s for the OC*H*_2_ and C*H*F_2_ resonance respectively at 11.7 T) when compared to ^19^F (7.2 s at 11.7 T). This acts to limit visible ^19^F magnetisation after direct transfer in the polarization transfer field.

Interestingly, the signal gains seen for this fluorinated alcohol were substantially lower than those of the fully protio counterpart for which ^1^H signal gains of *ca.* 500-fold have been previously reported.^41^ We attribute this change in part to modulation in the p*K*_a_ of the alcohol caused by introduction of the electronegative fluorine atoms. This is supported by the fact that very limited OH polarization is observed in the ^1^H NMR spectrum after SABRE-Relay. Use of even more acidic fluorinated alcohols such as hexafluoroisopropanol (p*K*_a_ = 9.3 (ref. 58)) result in no SABRE-Relay polarization being observed.

Similarly, the SABRE-Relay polarization of ^31^P nuclei is also achievable. When diethyl (hydroxymethyl) phosphonate was exposed to 3 bar *p*-H_2_ in the presence of ***d*_34_-2** and NH_3_, a 30-fold signal enhancement is observed for the ^31^P resonance after spontaneous polarization transfer at 70 G. Additionally, an 88-fold ^1^H signal gain for the OC*H*_2_ resonance was quantified. These signal gains are significantly reduced when compared to 1-propanol or 3-methyl-1-butanol and may be indicative of the alcohol being too acidic for SABRE-Relay with NH_3_. A further screen of less basic carrier amines may thus yield improved results.

## Conclusions

In summary, we have shown that the SABRE-Relay method can be used to transfer polarization from *p*-H_2_ to an alcohol *via* a polarization carrier amine. These results demonstrate how it is possible to broaden the scope of this hyperpolarization method to allow the rapid and cost effective detection of molecules present at low concentrations by magnetic resonance techniques. The SABRE-Relay effect is successfully mediated by the formation of an active polarisation transfer catalyst of the type [Ir(H)_2_(NHC)(amine)_3_]Cl and subsequent proton exchange between the hyperpolarised amine's NH and the target alcohol OH.

The amine plays a prominent role in this process and determines the size of the resulting signal gains seen in the nuclear spin orientation enhancement of the alcohol after SABRE-Relay transfer. Of the 24 amines investigated, NH_3_ performed best, yielding over 700-fold gains per proton in the OC*H*_2_ resonance of propanol. We conclude that in this case, the rate of proton exchange between NH_3_ and the alcohol most closely matches that needed for optimal NMR signal enhancement. The next best performing amine was *d*_7_-BnNH_2_ which, whilst exhibiting higher NH polarisation levels and longer relaxation times than NH_3_, led to alcohol NMR signal gains that were *ca.* 10% lower. Based on these data a match between carrier amine and target agent will be needed to deliver optimal SABRE-Relay performance. However, a role for isotopic labelling in the amine was exemplified with deuterated isotopologues yielding SABRE-Relay enhancements that were *ca.* 3 times higher than those of their protio counterparts. We attribute this to a reduction in the effects of polarisation transfer into the amine which leads to higher NH polarisation levels, alongside an increase in the NH relaxation time.

The effect of the magnetic field experienced by the sample during the SABRE-Relay transfer step was also examined over the range 0.5 to 140 G. It was shown that the largest signal gains were observed when this was set to *ca.* 70 G which corresponds to the point where the scalar couplings within the active SABRE catalyst must match optimally with PTF requirements for successful NH polarisation.^32^,^54^ This effect dominates even though a different field might be expected for subsequent spontaneous polarization transfer within the alcohol after proton exchange. This limitation is particularly evident when polarization is transferred from the OH into ^1^H, ^13^C, ^19^F or ^31^P nuclei in functionalised alcohols but could be circumvented in the future through the use of radio frequency driven transfer.^59^,^60^ Alternatively, this may open the door to the use of in-high-field methods such as LIGHT-SABRE.^61^–^63^

We were able to further improve on the initial polarisation levels by modulating the rate of ligand exchange during the SABRE process. Thus, by using a *tert*-butyl derived NHC ligand, the rate of amine dissociation from the active complex of type [Ir(H)_2_(NHC)(amine)_3_]Cl increased to 3.20 s^–1^ which results in improved NH signal gains and is followed through by improved NMR signal enhancements in the alcohol when compared to the [IrCl(COD)(IMes)] derived catalyst where the rate of loss is 1.64 s^–1^. When the amine dissociation rate is increased further by increasing the level of electron donation by ligands on the catalyst, the observed alcohol signal gains decrease. This supports previous evidence^34^,^43^ that the lifetime of the active SABRE complex governs the observed polarisation level and is shown here to be conveyed into the SABRE-Relay mechanism.

The results presented here have demonstrated that alcohols can be readily detected at concentrations as low as 2 mM using our SABRE-Relay conditions. However, at low relative equivalents of the target alcohol to the carrier amine the observed signal gains are reduced due to less efficient proton transfer. To overcome this and to further reduce the detection limit we propose that using a suitable co-ligand^64^,^65^ would enable the use of a substoichiometric amount of carrier amine. Thus, this would allow for a more efficacious ratio of amine to alcohol. For ^13^C detection the use of NH_3_ as a carrier is necessary and we have detected 3-methyl-1-butanol at a concentration of 14 mM in a single scan on a 9.4 T NMR system using an inverse probe. We expect this ^13^C detection limit can be improved upon by use and optimisation of ^1^H–^13^C INEPT pulse sequences. Low concentration detection of alcohols such as 3-methyl-1-butanol, which is present as an additive in many foods and drinks,^66^,^67^ demonstrates a potential role for SABRE-Relay in the detection of important low concentration analytes.

Given the ubiquitous nature of alcohols throughout chemical and biochemical literature, the results reported here could lead to the ability to gain further mechanistic insight into their reactivity by allowing the detection of low concentration species, whether as contaminants or intermediates. Finally, as the SABRE-Relay process expands the number of substrates amenable to *p*-H_2_ based polarisation this study may be useful in identifying a pathway to determine optimal conditions for the polarization of other functional groups.

## Conflicts of interest

There are no conflicts to declare.
